# Rat model of veno-arterial extracorporeal membrane oxygenation

**DOI:** 10.1186/1479-5876-12-37

**Published:** 2014-02-07

**Authors:** Ayyaz A Ali, Peter Downey, Gopal Singh, Wei Qi, Isaac George, Hiroo Takayama, Ajay Kirtane, Prakash Krishnan, Adrian Zalewski, Darren Freed, Stephen R Large, Euan A Ashley, Martin B Leon, Matthew Bacchetta, Ziad A Ali

**Affiliations:** 1 Center for Interventional Vascular Therapy, Division of Cardiology, New York Presbyterian Hospital and Columbia University, 161 Fort Washington Avenue, Herbert Irving Pavilion, 6th Floor, New York, NY 10032, USA; 2 Cardiovascular Research Foundation, New York, NY, USA; 3 Zena and Michael A. Weiner Cardiovascular Institute, Mount Sinai School of Medicine, One Gustav Levy Place, New York, NY 10029, USA; 4 Division of Cardiovascular Medicine, Stanford University, 870 Quarry Road, Stanford, CA 94305, USA; 5 Department of Cardiothoracic Surgery, Papworth Hospital and University of Cambridge, Cambridge, CB23 3RE, United Kingdom; 6 University of Alberta and Mazankowski Alberta Heart Institute, 8440 112 St NW, Edmonton, AB T6G 2P4, Canada

**Keywords:** ECMO, Extracorporeal membrane oxygenation, Resuscitation, Cardiac arrest

## Abstract

**Background:**

We aim to develop a rat model of veno-arterial extracorporeal membrane oxygenation (VA-ECMO).

**Methods:**

VA-ECMO was established in twelve Male Sprague-Dawley rats (250-350 g) through cannulation of the right jugular vein for venous drainage and the right femoral artery for arterial reinfusion. Arterial blood pressure was measured using a conductance catheter through cannulation of the left carotid artery. Heart rate was monitored by electrocardiography and arterial blood gas parameters with a blood gas analyzer. The VA-ECMO circuit was tested by subjecting the rats to hypoxic cardiac arrest with resuscitation using VA-ECMO. Both load-dependent and load-independent measures of myocardial contractility were measured using pressure-volume loop analysis to confirm restoration of myocardial function post-resuscitation.

**Results:**

Following hypoxic cardiac arrest VA-ECMO provided sufficient oxygenation to support the circulation. The haemodynamic and blood gas parameters were maintained at transition and during ECMO. All animals were resuscitated, regained cardiac function and were able to be weaned off ECMO post-resuscitation.

**Conclusion:**

We have established a safe, high-throughput, economical, functioning rat model of VA-ECMO.

## Background

Extracorporeal Membrane Oxygenation (ECMO) is a type of mechanical circulatory support that is used for resuscitation of patients with failing pulmonary and cardiac function [1]. However, no effective rodent models of ECMO support following cardiac arrest currently exist to allow evaluation of post-arrest myocardial function. We describe a novel technique for instituting veno-arterial extracorporeal membrane oxygenation (VA-ECMO) in a rodent for resuscitation following hypoxic cardiac arrest. This method restores the native circulation allowing the opportunity to conduct further studies on the physiological and molecular changes occurring in the myocardium during and after cardiac arrest.

## Methods

All animals received humane care in compliance with the 'Principles of laboratory animal care’ formulated by the National Society for Medical Research and the 'Guide for the care and use of laboratory animal resources’ published by the US National Institute of Health (NIH publication No. 85-23, revised 1996). The following studies were approved by the Columbia University Institutional Animal Care and Use Committee (IACUC) under protocol AC-AAAG6406 and conform to the Guide for the Care and Use of Laboratory Animals published by the National Institutes of Health.

### Animals

Twelve 250-350 g male Sprague-Dawley rats were originally sourced from Charles River Laboratories for use in the experiments. Rats were maintained in temperature-controlled (20°C to 22°C) cages with a 12-hour light-dark cycle, and received free access to sterilized water and standard rodent chow (Rodent diet BK002P, B & K Ltd).

### Anesthesia and ventilation

Rats were anesthetized using 3-4% isoflurane in an induction chamber and maintained at 1.5-2.5% through a nosecone. Endotracheal intubation was undertaken with a 14G cannula (Smiths Medical, London, UK).

### Operative procedure

The operative procedure, including implantation of all ECMO cannulae, was conducted in the live animal, prior to the induced hypoxic cardiac arrest. The animal was positioned with the lower limbs closest to the operating surgeon. Under visualization of a dissecting microscope an incision was made in the right groin to access the femoral vessels. The femoral artery was dissected free from the femoral vein, and the femoral vein was cannulated for administration of fluids and medication. One-hundred units of intravenous heparin were administered for anticoagulation.

A median sternotomy was performed to allow direct visualization of the heart for confirmation of cardiac arrest. The sternum was divided with electrocautery taking caution to avoid the right and left internal thoracic arteries. The chest was retracted open with a miniature Finochietto retractor and the thymus removed with electrocautery to facilitate visualization of the heart, lungs, and great vessels.

**Figure 1:**
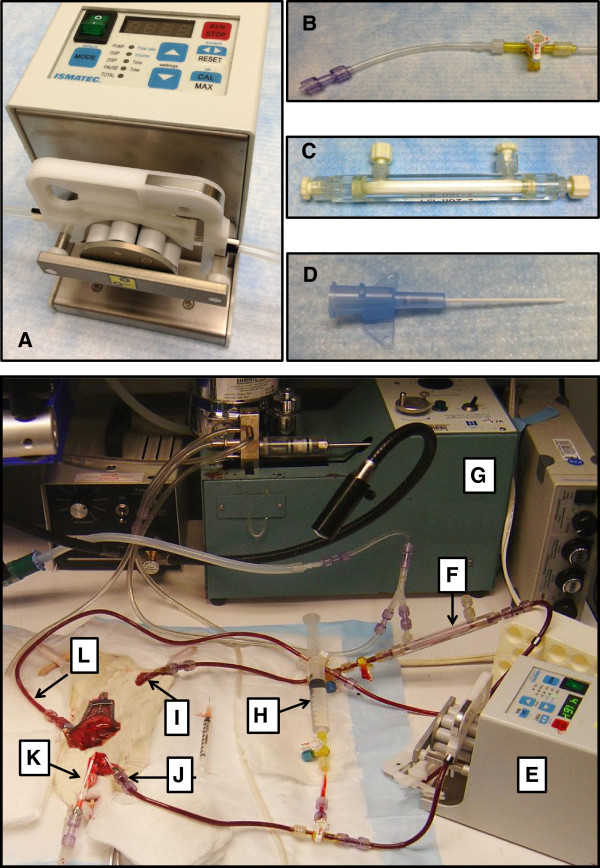
**VA-ECMO circuit.** Roller pump **(A)**, tygon tubing conduit with three-way stopcock and male-male connector **(B)**, silicone membrane oxygenator **(C)**, 24 G femoral arterial cannula **(D)**. Bottom: Ismatec roller pump **(E)**, silicone membrane oxygenator **(F)**, small animal ventilator **(G)**, reservoir **(H)**, right femoral artery cannula **(I)**, right internal jugular vein cannula **(J)**, endotracheal tube **(K)**, left ventricular venting catheter **(L)**.

For ECMO inflow, one centimetre of femoral artery was exposed from the inguinal ligament distally. A small hemostatic clamp (Yasargil, Germany) was applied as proximal as possible to occlude the femoral artery and the distal end of the femoral artery ligated. A small arteriotomy was made in the femoral artery and a 24 G angiocath connected to 4 mm tygon tubing primed with normal saline (Figure 1D) was advanced into the femoral artery. The cannula was secured with two 6.0 nylon ligatures. The clamp previously placed on the femoral artery was removed. In some animals a left ventricular venting catheter was placed to prevent overdistention.

For ECMO outflow the right jugular vein was dissected in the cervical region and a small hemostatic clamp applied proximally to occlude the jugular vein. The vessel was then ligated and retracted as distal as possible above the clavicle to allow for cannulation. A small venotomy was made at the distal end of the jugular vein and a 20 G angiocath cannula connected to 4.0 mm tygon tubing advanced into the vein. A ligature was loosely placed around the jugular vein to secure the cannula and then the clamp occluding the vessel was removed. The cannula was then gently manipulated until it was positioned in the superior vena cava (SVC) under direct visualization.

### Haemodynamic monitoring

Continuous acquisition of haemodynamic data was used to complement the procedure and allow for precise monitoring of arterial pressure before and after ECMO reperfusion. The right common carotid artery was dissected in the cervical region. The vessel was ligated as distally in the neck as possible, and a small occlusive clamp (Yasargil, Germany) was used to occlude the carotid artery proximal to the site of intended cannulation. A Millar ultra miniature pressure catheter was connected to a Millar-Ultra PVS console for measurement of left ventricular pressure over the course of the experiment. A small arteriotomy was made in the carotid artery distal to the clamp and the catheter was inserted. A 6.0 ligature was used to secure the catheter within the vessel.

### Establishment of the extracorporeal membrane oxygenation circuit

The equipment and our ECMO model are depicted in Figure 1. A micro-peristaltic pump was utilized for propulsion within the circuit (Digital Reglo Pump, Ismatec, Ltd; Figure 1A, 1E). This is a digital tubing pump with 8 rotors. The pump is marketed for flows ranging from 0.002-57 ml/min with rotor speeds ranging from 1.6 to 160 revolutions per minute. The required prime volume for our ECMO model is 8 ml normal saline. 4.0 mm tygon tubing was inserted into a customized pump cassette (Ismatec, Ltd) and the outflow tubing arm was connected to a custom miniature oxygenator device (Figure 1C, 1F). Heparinized saline was used to prime the oxygenator and the circuit tubing. The pump inflow tubing was connected to the venous drainage cannula and the oxygenator was then connected to the arterial infusion cannula (Figure 1D). Finally a 12 ml syringe was added into the circuit proximal to the pump via a 3-way stopcock (Figure 1H) to act as a reservoir for addition of fluid if necessary to support pump flow, which can occur with changes in position of the venous cannula within the SVC. Arterial blood gases were measured using an Epoc blood analysis device (Epocal Inc, Ontario, Canada). The parameters of haemodynamics and blood gas analysis were recorded at 3 time points: preoperatively (0 mins), post cardiac arrest (15 mins), post resuscitation (60 min). Additional blood gas analysis were performed ad hoc post resuscitation to ensure correction of metabolic acidosis.

### Statistics

Data were subjected to the Kolmogorov-Smirnov test to determine distribution. Descriptive variables are presented as means ± SD and compared with the *t*-test. When comparing multiple groups data was analysed by analysis of variance with Bonferroni post-test for multiple comparison of parametric data. Estimations are presented with 95% confidence intervals. Conventional levels of significance (0.05) were applied throughout. Statistical analysis was undertaken using SPSS for windows version 19 (Chicago, Ill) and S Plus version 6 (Seattle, Wash).

## Results

### Cardiac arrest

**Figure 2:**
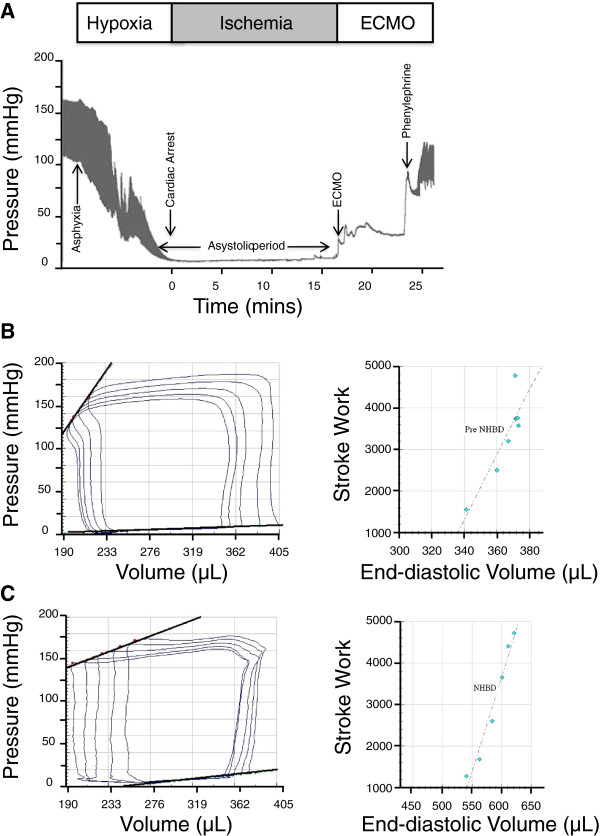
**Haemodynamics and myocardial contractility pre and post VA-ECMO resuscitation of hypoxic cardiac arrest.** Phases of the experiment showing blood pressure changes during hypoxia, ischaemia, and VA-ECMO resuscitation **(A)**. Pressure-volume loops pre-arrest **(B)** and post VA-ECMO resuscitation **(C)** showing an impairment in the end-systolic pressure volume relationship (upper slope of the PV loops) but preserved pre-load recruitable stroke work.

We conducted experiments to evaluate the cardiac function of the rat heart after post-circulatory arrest with warm ischaemia and subsequent reperfusion. Once all ECMO cannulae were successfully implanted, we induced hypoxic cardiac arrest by clamping the trachea to produce asphyxiation. Tracheal occlusion was followed by progressive hypotension and bradycardia leading to pulseless electrical activity cardiac arrest. Following a period of circulatory arrest with 15 minutes of warm ischaemia, the animals were reperfused with normothermic oxygenated blood using VA-ECMO (Figure 2A). We initiated flow at the lowest pump setting (0.001 ml/min) and gradually increased flow until the arterial perfusion pressure was approximately 25-30 mm Hg as measured by the pressure-volume loop catheter. There was some variability in the flow rate required to maintain these parameters in each animal, but in general, flow ranged from 5-6 ml/min. Reperfusion was followed by the development of ventricular fibrillation and subsequently spontaneous cardioversion into normal sinus rhythm at a mean of 7.3 ± 2.8 minutes.

Upon return of sinus rhythm, ECMO reperfusion resulted in an increase of 24 ± 4.2 mmHg in the mean arterial pressure (mAP). The period of warm ischaemia led to significant metabolic abnormalities (Table 1) including severe acidosis (pH 7.0 ± 0.1) that were corrected with bolus injections of 500 μL 8.4% sodium bicarbonate assessed by repeat arterial blood gases. Administration of 0.1 ml of 10 mg/mL phenylephrine augmented mAP, accelerating correction of acidosis that further increased mAP to 52 ± 6.3 mmHg. As the mAP improved, the ECMO flow rate was reduced over 5 minutes and mechanical ventilation/oxygenation restarted while continuing ECMO support for another 30 minutes. After 30 minutes, VA-ECMO could be completely stopped and the resuscitated heart was able to sustain the circulation independently in all animals and metabolic homeostasis was achieved independent of further pharmacological correction. No animals required more than one attempt at weaning ECMO support to demonstrate adequate cardiac function to maintain unsupported perfusion.

**Table 1:** Metabolic changes pre and post VA-ECMO resuscitation of hypoxic cardiac arrest

**Biochemical parameter**	**Baseline**	**Anoxia**	**Resuscitation**	**P**
HCO3-, mmol/L (SD)	20.9 (0.8)	14.1 (2.2)*	19.6 (1.8)	<*0.001*
pH, (SD)	7.49 (0.1)	6.99 (0.1)*	7.38 (0.1)	<*0.001*
pCO_2,_, mmHg (SD)	23 (11)	105 (29)*	42 (8)	<*0.001*
pO_2,_, mmHg (SD)	225 (37)	64 (17)*	270 (55)	<*0.001*
Hb, g/dl (SD)	15.5 (1.2)	16.4 (1.1)	9.2 (2.1)*	<*0.001*
HCT, % (SD)	47 (4)	50 (3)	31 (3)*	<*0.001*
sO_2_, % (SD	96 (2)	48 (18)*	99 (3)	<*0.001*
K+, mmol/l (SD)	3.7 (0.8)*	5.5 (1.0)	5.1 (1.2)	<*0.001*
Ca+, mmol/l (SD)	2.5 (0.8)	2.4 (0.3)	2.2 (0.4)	0.73
Lactate, mmol/L (SD)	3.3 (0.5)	3.7 (1.1)	2.8 (0.9)	0.43

HCO_3_-: Bicarbonate; *pCO*_2_: partial pressure of CO_2_; pO_2_: partial pressure of O_2_; *Hb*: Hemoglobin; *HCT*: Hematocrit; sO_2_; K+: Potassium; Ca+: Calcium. Continuous data are presented as mean with 95% confidential intervals (95% CI) or as median with interquartile ranges (IQR 25-75). *Bonferroni’s Multiple Comparison Test.

### Assessment of haemodynamic and contractile function

Using micro-conductance catheters we obtained pressure-volume (PV) loops from the left ventricle (LV) of experimental animals. Pre-cardiac arrest and post VA-ECMO resuscitation data are presented in Table 2. Compared to pre-arrest (Figure 2B), following resuscitation load independent measures of contractility demonstrated a reduced end-systolic pressure volume relationship and end-systolic stiffness (E_es_ E_max_), yet pre-load recruitable stroke work was sustained (Figure 2C). Load dependent measurements demonstrated a significant worsening of dP/dt max and min in the VA-ECMO resuscitated heart but no significant difference in cardiac output, stroke volume or stroke work. Diastolic function assessed by Tau was also impaired in the ECMO resuscitated heart (Table 2).

**Table 2:** Measures of myocardial contractility pre and post VA-ECMO resuscitation of hypoxic cardiac arrest

***Load independent measurements***	**Baseline**	**Post**-**arrest**	**P**
E_es_ (E_max_)_,_ mmHg/μL (SD)	2.3 (0.8)	1.4 (0.9)	*0.04*
PRSW, mmHg (SD)	60 (22)	70 (30)	0.46
***Load dependent measurements***			
dP/dt max, mmHg/s (SD)	5221 (826)	3196 (1045)	<*0.001*
dP/dt min, mmHg/s (SD)	-5141 (1176)	-2162 (542)	<*0.001*
Cardiac output, μl/min (SD)	25749 (13835)	27008 (6994)	0.92
Stroke volume, μl (SD)	95 (38)	147 (51)	0.11
Stroke work, mmHg/μl (SD)	7192 (2566)	7371 (3419)	0.9
***Diastolic function***			
Tau Glantz, msec (SD)	11.5 (2.0)	17.7 (8.4)	0.07
EDPVR (SD)	0.03 (0.0)	0.04 (0.03)	0.42

*E*_*es*_ (E_max_), end-systolic elastance (slope of the end-systolic relationship); Preload recruitable stroke work; dP/dt max: Maximum rate of LV pressure change during systole; dP/dt min: Minimum rate of LV pressure change during systole; *EDPVR*: End diastolic pressure volume relationship. Continuous data are presented as mean with 95% confidential intervals (95% *CI*) or as median with interquartile ranges (IQR 25-75).

## Discussion

Well-functioning rat models of cardiopulmonary bypass have been established, including recent examples that do not require priming with blood [2]. While these models are helpful, ECMO is fundamentally different than cardiopulmonary bypass in its clinical applications. The full bypass circuit is not applicable to the patient who needs long-term cardiopulmonary support. ECMO is applicable to a broad range of clinical scenarios, including bedside cannulation for support during instances of hypoxic cardiac arrest [3,4]. A rabbit model of ECMO has recently been described to test these applications [5]. Similar to the rabbit model, rats have a nearly identical anatomy to humans with the advantage of being smaller, less expensive, and easier to handle than larger animal models.

A small animal model for ECMO is necessary because it provides an efficient, economical, and accurate model for the study of physiologic changes during ECMO. The estimated cost of equipping a laboratory with a basic microsurgical setup such as dissecting microscope, small animal ventilator, and surgical instruments is approximately $3000. The costs specifically related to the ECMO circuit include micro-oxygenators ($250 each/4-6 uses), Tygon tubing ($200), intravenous cannuale ($200) and the micro-peristaltic pump ($2400). In our model, nearly all equipment is reusable and the cost incurred per experiment is largely related to the purchase and boarding costs of the rat (Male Sprague-Dawley rat, $30, Charles River Laboratories, Cambridge, MA; Boarding, approximately $20 for acclimatization and prompt usage).

In our study, we developed the first rodent model of VA-ECMO that was used for successful resuscitation following an induced hypoxic cardiac arrest. After a 30-minute period of supportive reperfusion, ECMO was weaned to assess the ability of the resuscitated heart to support the circulation independently. Based on our results, we were able to show that this model allows for return of cardiac function and serves as the basis for subsequent investigation in areas such as post-myocardial arrest depression, cardiogenic shock, hemodynamic support and cardiovascular resuscitation.

## Conclusions

We have established a reliable and economical rat model for VA-ECMO. Biochemical parameters and pressure-volume relationships show that despite induced hypoxic cardiac arrest, our VA-ECMO circuit was capable of restoring adequate circulation for return of full cardiac function and perfusion.

## Abbreviations

ECMO: (Extracorporeal membrane oxygenation); VA-ECMO: (Veno-arterial extracorporeal membrane oxygenation); NIH: (National Institutes of Health); IACUC: (Institutional Animal Care and Use Committee); SVC: (Superior vena cava); PV: (Pressure-volume).

## Competing interests

The authors declare that they have no competing interests.

## Authors’ contributions

AA was largely responsible for the overall concept, design, and data acquisition of this study. PD participated in the experiments and assisted in the creation of this manuscript, and made substantial contributions to drafting and revising this article for important intellectual content. GS participated in the experimental procedures. IG assisted in the interpretation of data. HT, AK, and PK participated in the review of the experimental design and the review of this manuscript. AZ assisted with the experiments and helped create the figures for this manuscript. EA and SL offered important scientific guidance in the experimental design and the interpretation of the data. DF contributed significant advice on surgical technique. MBL reviewed and approved the final versions of this article in preparation for submission. ZA oversaw this project as a whole, reviewed all data analysis and made important revisions of the manuscript for submission. All authors read and approved the final manuscript.
